# Supplementary material for: Splice-Junction-Based Mapping of Alternative Isoforms in the Human Proteome
Source: Cell Rep. Author manuscript; Available in PMC 2020 Jan 15. (PMC6961840; doi:10.1016/j.celrep.2019.11.026)
Supplement: 2 [file NIHMS1546469-supplement-2.zip › DF1/PXD010154/Liver/Liver_16_SRSF11_VTAQPDVLEVQAEYITAGPGPSGGPGGGG.pdf]

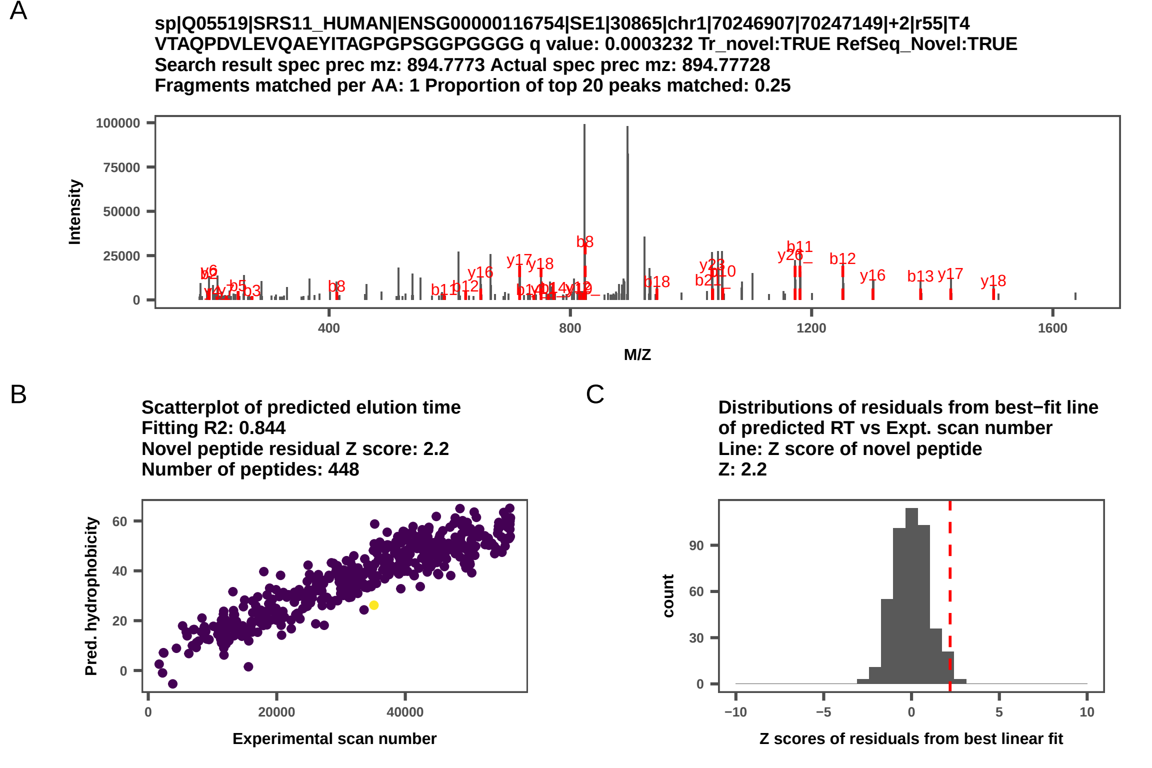
A
sp|Q05519|SRS11_HUMAN|ENSG00000116754|SE1|30865|chr1|70246907|70247149|+2|r55|T4
VTAQPDVLEVQAEYITAGPGPSGGPGGGG q value: 0.0003232 Tr_novel:TRUE RefSeq_Novel:TRUE
Search result spec prec mz: 894.7773 Actual spec prec mz: 894.77728
Fragments matched per AA: 1 Proportion of top 20 peaks matched: 0.25
Intensity
100000
75000
50000
25000
0
400
800
1200
1600
M/Z
b8
b11
y26
b12
y16
b13
y17
y18
y23
b21
b10
y18
y16
y17
y18
b8
b5
b3
B
Scatterplot of predicted elution time
Fitting R2: 0.844
Novel peptide residual Z score: 2.2
Number of peptides: 448
Pred. hydrophobicity
60
40
20
0
0
20000
40000
Experimental scan number
C
Distributions of residuals from best−fit line
of predicted RT vs Expt. scan number
Line: Z score of novel peptide
Z: 2.2
count
90
60
30
0
−10
−5
0
5
10
Z scores of residuals from best linear fit
